# Identification of Serum Biomarkers for Differentiating Epileptic Seizures from Psychogenic Attacks Using a Proteomic Approach; a Comparative study

**Published:** 2020-10-29

**Authors:** Mohsen Parvareshi Hamrah, Mostafa Rezaei Tavirani, Monireh Movahedi, Sanaz Ahmadi Karvigh

**Affiliations:** 1 Department of Biochemistry, Faculty of Biological Science, North Tehran Branch, Islamic Azad University, Tehran, Iran.; 2 Proteomics Research Center, School of Allied Medical Sciences, Shahid Beheshti University of Medical Sciences, Tehran, Iran.; 3 Department of Neurology, Sina Hospital, Tehran University of Medical Sciences, Tehran, Iran.

**Keywords:** Epilepsy, Proteomics, Biomarkers, Diagnosis, Differential, Emergency Service, Hospital

## Abstract

**Introduction:**

Differentiating actual epileptic seizures (ESs) from psychogenic non-epileptic seizures (PNES) is of great interest. This study compares the serum proteomics of patients diagnosed with ESs and PNES.

**Methods:**

Eight patients with seizure (4 with PNES and 4 with TLE (temporal lope epilepsy)) were enrolled in this comparative study. Venous blood samples were drawn during the first hour following the seizure. Standard protein purification technique was employed and proteins were subsequently separated via 2-D electrophoresis. After comparison of the serum proteomes from the two groups, protein expression was analyzed. The differentially expressed bands were determined using both matrix-assisted laser ionization time-of-flight (MALDI/TOF) and electrospray ionization quadruple mass spectrometry (MS).

**Results:**

This study identified 361 proteins, the expression of 110 proteins increased, and 87 proteins decreased in the PNES group compared with TLE group. Four separate proteins were finally identified with MALDI/TOF MS analysis. Compared with PNES group, alpha 1-acid glycoprotein, ceruloplasmin, and S100-β were down-regulated and malate dehydrogenase 2 was up-regulated in the serum of TLE patients.

**Conclusion:**

Our results indicated that changes in serum levels of S100-β, ceruloplasmin, alpha 1-acid glycoprotein 1, and malate dehydrogenase 2 after seizure could be introduced as potential markers to differentiate ES from PNES; however, more advanced studies are required to reach a better understanding of the underlying mechanisms.

## Introduction

Psychogenic non-epileptic seizures (PNES) neither cause changes in paroxysmal behaviors, nor make changes in ictal, peri- and inter-ictal electroencephalography (EEG), both of which are characteristics of epileptic seizures (ESs) (1). Temporal lobe epilepsy (TLE), the most common type of epilepsy in humans, is frequently associated with hippocampal sclerosis, cognitive deficit, and pharmaco-resistant seizures (2, 3). Up to 80% of the patients who have been diagnosed with PNES via video-electroencephalography (vEEG) were at least on one anti-epileptic drug (AED) at the time of diagnosis (4). Around 5–20% of patients admitted to epilepsy wards to be monitored with vEEG are diagnosed with PNES and 20-30% of patients with intractable ESs are also differentially diagnosed with PNES (5). Misdiagnosis results in years of improper medical treatment, which apart from the financial burden affecting their social life, could cause loads of undesirable and sometimes even unbearable side-effects in patients (6-8). The gold-standard approach to diagnose psychogenic non-epileptic seizures (PNES) is vEEG, in which a typical ES episode in absence of any change in the ictal tracing is recorded. But the technique has its own limitations; it demands prolonged hospitalization, is not available in many countries, most patients cannot afford it, and is not always able to recognize the seizure episode. Therefore, globally, scientists are testing other diagnostic approaches to differentiate ESs from PNES. In this regard, various diagnostic biomarkers are gathering more and more attention. Comparative proteomic analysis is a powerful diagnostic tool by which the onset, progression, and prognosis of the diseases could be determined in humans. It is especially helpful when the mechanism of a biological process is being investigated, since the technique makes it possible to simultaneously identify a large number of proteins and their alterations, each of which corresponds to a certain pathological condition. The present study aimed to identify differentially expressed proteins, which can differentiate ESs from PNES. 

## Methods

***2.1. Study design and setting***

In this comparative cross-sectional study, out of all patients with prior history of recurrent seizures, admitted to epilepsy monitoring unit (EMU) of Sina Hospital, Tehran, Iran, a total of 8 patients (4 patients suffering from TLE and 4 patients suffering from PNES) were enrolled. After comparison of the serum proteomes from the two groups, protein expression was analyzed and differentially expressed bands were determined. Written informed consent was obtained from all patients before entering the study. All procedures performed on human subjects in this study were in accordance with the ethical standards of the institutional and/or national research committee and with the 1964 Helsinki Declaration and its later amendments or comparable ethical standards. Additionally, the ethics committee of Shahid Beheshti University of Medical Sciences approved of this study under the registration number IR.SBMU.RETECH.REC.1397.289.

**2.2. Data gathering**

 The variables considered for data collection were patient's age, sex, duration and frequency of epilepsy, related risk factors, and received medications. The exclusion criteria of the study included using psychoactive drugs or medications other than antiepileptic drugs (AED), and having neurologic or psychiatric diseases or a recent brain trauma.

In order to obtain habitual events, Video-EEG (VEEG) monitoring was performed for all patients. A skilled epileptologist identified the type of epilepsy considering ictal and interictal EEG results and the semiology of the seizures. 

**2.3. Sample preparation**

Blood samples were drawn within 1 hour following seizure episodes in patients. Samples were centrifuged at 4000 rpm for 10 minutes and serum aliquots were kept at −80 °C until needed. 

**2.4. Proteomic experiment**

2D-electrophoresis materials used were made by GE HealthCare Life Sciences (http:// www.gelifesciences.com) and SERVA Company (http://www.serva.de). Pooling was performed for the two groups, separately, and protein extraction was done using 2-DE Clean-Up Kit (GE Healthcare). The concentrations of the protein were reported using 2-DE Quant Kit (GE Healthcare) and then 2-DE performed on triple replicate samples. The first dimension, Isoelectric Focusing (IEF), separates proteins based on their isoelectric point (pI). Before this phase, Immobilized pH gradient (IPG) strips were passively rehydrated for 8 hours. Then, Isoelectric Focusing (IEF) was carried out using Bio-Rad PROTEAN IEF Cell, 11 cm nonlinear IPG with pH range 4-7 for 7.5 hours at 20 °C, according to the Bio-Rad Protocol. After that, the IPG strips were equilibrated in equilibration solution (Serva Kit) for 30 minutes at room temperature. The next step consisted of separation of the proteins based on Molecular Weight (MW) via HPE Flattop Tower (horizontal electrophoresis) using 2D HPE™ Double-Gel 12.5 % Kit (Serva Company) for 3.5 hours. After electrophoretic separation of the proteins, gels were stained using SERVA HPE™Coomassie® Staining Kit according to the manufacturer's instructions. Stained gels were then scanned using a calibrated GS-800 densitometer scanner (Bio-Rad) (9). Protein expression of the two samples was quantified and qualified using Progenesis Same Spots software as an image analyzer. 

***Statistical analysis***

The cut-off point was considered 1.5-fold increase or decrease. Statistically significant differences (P≤0.05) in spot intensities were determined via one-way ANOVA analysis as a multivariate statistical analysis tool analyzing gel images using the alignment method. The criteria for gel analysis were 1.5-fold change. Finally, using MALDI-TOF MS, the candidate spot was evaluated. Protein spots before treatment with trypsin, were de-stained and treated with dithiothreitol (DTT) and iodoacetamide for reduction and alkylation, respectively. In the end, to identify the protein, the extracted peptides were assessed by MS and the spectra were entered into MASCOT (http://www.matrixscience.com).

## Results

**3.1. Demographic features of different study groups**

V-EEG showed that 4 patients (2 male and 2 female) with the mean age of 37.5 ± 7.76 years had mesial TLE and 4 patients (2 male and 2 female) with the mean age of 33.25 ± 18.7 years had PNES. Patients in PNES group experienced seizures with the frequency range of 3-30 per month, while the frequency range of seizure experience for the patients in ES group was 2-4 episodes per month. The duration of the disease was 3-12 years in PNES group and 4-24 years in ES group. All members of the study population were on multidrug therapy. Two patients (one in PNES group and one in ES group) were on psychiatric medications (antidepressants or neuroleptics). 

**3.2. Proteomic analysis**

**Figure 1:**
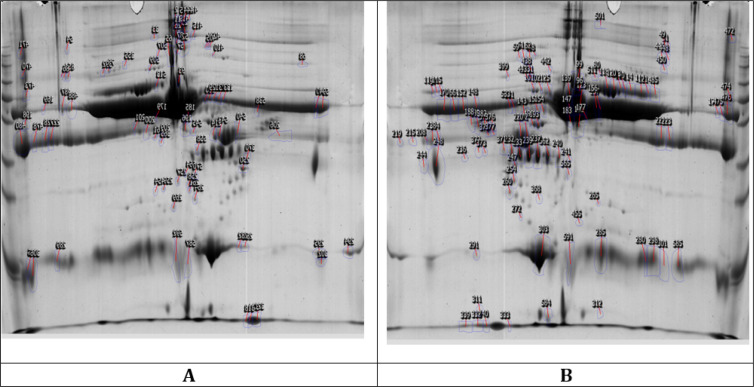
Representative two-dimensional electrophoresis gels of serum proteins of patients with temporal lobe epilepsy (A) and psychogenic non-epileptic seizures (B).

Serum analysis of ES group (Figure 1A) and PNES group (Figure 1B) identified alteration in the expression of 197 proteins out of the total of 361 identified proteins, 110 (55.9%) of which showed up-regulation, and 87 (44.1%) showed down-regulation in the PNES group when compared to ES group. As summarized in Table 1, four distinct proteins (S100-β, malate dehydrogenase 2, alpha acid 1-glycoprotein 1, ceruloplasmin) were identified through MALDI/TOF MS analysis. The expression of alpha acid 1-glycoprotein 1, ceruloplasmin, S100-β had decreased in the serum of patients in ES group, and malate dehydrogenase 2 had increased in ES group. Table 2 demonstrates the identified proteins’ principal cellular and molecular functions.

**Table 1:** Differentially expressed proteins in the serum of patients with temporal lope epilepsy (TLE) compared to psychogenic non-epileptic seizures (PNES) group

Spot No.	Gene Name	UniProt Code	pI/Mw (Da)1	pI/Mw (Da)2	Fold	Regulation*
311	S100-β	P04271	4.57/10.713	4.84/10	3.5	Up
258	MDH2	P40926	8.92/35.503	6.52/22	2.3	Down
248	ORM1	P02763	4.93/23.52	3.93/25	2.3	Up
59	CP	P00450	1.065/5.44	5.67/140	2.3	Up
61	CP	P00450	1.065/5.44	5.73/128	5.3	Up
62	CP	P00450	1.065/5.44	5.81/128	2.8	Up

1: Theoretical; 2: Observed; *: in PENS group; MW: Molecular weight; pI: Isoelectric point; Da: Dalton MDH2: Malate Dehydrogenase 2; ORM1: Alpha 1- Acid Glycoprotein 1; CP: Ceruloplasmin.

**Table 2:** Cellular and molecular functions of identified proteins

**Protein name**	**Function**
S100-β	Calcium ion binding; calcium-dependent protein binding; identical protein binding; protein binding; protein homo dimerization activity; RAGE receptor binding; S100 protein binding; tau protein binding; zinc ion binding
MDH2	L-malate dehydrogenase activity; malate dehydrogenase (NADP+) activity; protein self-association
ORM1	Protein binding
CP	Chaperone binding; copper ion binding; ferroxidase activity

The function of each protein was identified using the UniProt database. MDH2: Malate Dehydrogenase 2; ORM1: Alpha 1- Acid Glycoprotein 1; CP: Ceruloplasmin.

## Discussion

Our results showed significant differences between the serum levels of four proteins including ceruloplasmin, malate dehydrogenase 2 (MDH2), S100B, and alpha-1-acid glycoprotein 1 in ES and PNES groups, which can be considered as suitable biomarkers for distinguishing PNES from ES. Over the past decades, a wide variety of diagnostic approaches have been developed to identify PNES, leading to introduction of electroencephalography (vEEG) as the gold standard method to differentiate PNES from ES (10). Despite the favorable results, using this approach is associated with some challenges, including high cost and lack of availability for all clinical centers. Moreover, vEEG assessment only confirms the epileptic discharge in patients suffering from ES, whereas it is not a confirmation test for those with PNES (1, 11). A glance at the misdiagnosed PNES rate reveals the importance of definitive diagnosis of true PNES cases. It is reported that about 20% of patients with manifestations of refractory epilepsy have PNES. Moreover, approximately 30% of cases that are diagnosed as refractory epilepsy and referred to surgery suffer from PNES (12). Thus, the searches for new biological and psychological markers to identify PNES cases continues. In this light, using blood biomarkers for diagnosis of PNES is of interest to researchers because of their low cost and sample availability. Unfortunately, despite the extensive efforts in this context, no reliable biomarker has been discovered to certainly distinguish PNES from the other types of seizure. To date, various biomarkers such as cortisol (13, 14), prolactin (PRL) (15, 16), neuron-specific enolase (NSE) (17, 18), brain-derived neurotrophic factor (BDNF) (19), and Ghrelin and Nesfatin-1 (20) have been discussed as potential markers to differentiate ES from PNES. Among them, PRL showed to have the highest sensitivity. Measuring serum level of PRL within 20-30 min after the seizure has been recommended by The American Academy of Neurology (AAN) as a helpful test to differentiate ES from PNES (17, 20-22). It is important to measure PRL levels as early as possible as it returns to baseline 2-6 hours after the seizure (23). According to a study done by D’Alessio et al. measuring PRL after seizure had the sensitivity and specificity of 69% and 93% in differentiation of ES from PNES, respectively (15). High serum PRL levels should be taken seriously, as it is susceptible to alteration by different physiological and pathological conditions, and different medications (23). Serum creatinine kinase (CK) is one of the elements whose level after seizure has been considered medically related to differentiating ES from PNES (sensitivity of 14.6% to 87.5% and specificity of 85% to 100%) (24, 25). It is important to mention that increased serum CK levels should also be taken seriously, as many different clinical complications could cause it (26, 27). In recent years, progress in the field of proteomics has opened a promising window to find new candidates in the context. In the present study, serum proteins of patients suffering from ES and PNES were compared. It is important to state that the difference in protein expression in the two gels can help us identify protein markers that are vital for diagnosis and treatment of related diseases. In our previous effort, up-regulated proteins with higher molecular weights and high isoelectric point were reported to be present in patients with temporal lobe epilepsy (TLE) compared with PNES samples. Our study showed that a critical event that can be used for discriminating PNES from ES is damage to blood brain barrier in epileptic seizure. Thereby, to have a thorough view of ES, the deregulated proteins need to be identified (28).

Ceruloplasmin is a ferroxidase enzyme that belongs to a multicopper oxidase family. This enzyme possesses three copper sites in its structure and carries the major part of copper ions (>95%) in plasma. Ceruloplasmin is also involved in iron metabolism (29). Our findings indicated that patients suffering from PNES had a higher amount of ceruloplasmin compared to those with ES. So far, many studies have shown the differences in Cu^2+^ concentration among patients with ES (30, 31). For instance, Hamed et al. found that the serum concentration of Cu^2+^ in untreated-ES patients was significantly higher than patients receiving anticonvulsant drugs (32). However, no study has compared the serum levels of ceruloplasmin in patients suffering from ES and PNES. According to our result, the higher concentration of ceruloplasmin in patients with PNES gives a hint for further investigations on the serum levels of Cu^2+ ^for differential diagnosis of PNES from ES. 

For the first time in literature, we found the difference between MDH2 levels in the serum samples of patients with ES and PNES. Our results showed a higher amount of MDH2 in patients with epilepsy compared with those suffering from PNES. It is reported that MDH2 is a negative regulator of *SCN1A *gene expression at the posttranscriptional level (33). *SCN1A *gene encodes the Voltage-gated sodium channel α-subunit type I protein (NaV1.1) that plays an important role in brain excitability. A wide variety of studies have confirmed the correlation between the down-regulation of *SCN1A *and epilepsy (34, 35). Chen et al. reported that MDH2 directly binds to the 3′ UTR region of SCN1A mRNA, which decreases the mRNA stability. Accordingly, the forced overexpression of MDH2 in HEK-293 cells resulted in down-regulation of SCN1A expression. They also found that there was an inverse correlation between the amount of *NaV1.1* and MDH2 proteins in the hippocampus of seizure mice (33). This finding suggests the potential of MDH2 as a predictive biomarker of epilepsy.

S100-B protein is mainly produced by astrocytes that are involved in diverse biological events such as cell proliferation, differentiation, apoptosis, and migration (36, 37). This Ca^2+ ^binding protein exhibits characteristics that make it a suitable candidate to be used as a biomarker of brain damages (38). Increase in the serum level of S100-B in patients with epilepsy and infants suffering from epileptic seizures has revealed the high potency of this protein for clinical diagnosis of epilepsy (39, 40). It has been suggested that S100B protein plays a part in the anti-epileptogenic effect of astrocytes. Following the electrical kindling of the amygdala, Dyck et al. reported that S100B-knockout mice had more rapid and severe seizures compared with normal mice (41). In a study conducted by Chang et al., the serum levels of S100B in thirty-four patients with temporal lobe epilepsy (TLE) were analyzed. In their study, S100B serum level in patients with more than two seizures per month was significantly higher than those with less than two seizure episodes per month (42). However, using S100-B protein as an epilepsy biomarker is controversial and some studies have reported no significant differences between the serum levels of S100ß in patients with TLE and controls (43, 44). Recently, Asadollahi et al. evaluated the serum level of S100-B in patients with epilepsy, PNES, and healthy individuals. According to their findings, S100-B was significantly higher in patients suffering from ES compared with those with PNES as well as healthy controls. On the other hand, patients with PNES showed a higher concentration of S100-B in their sera compared with controls (45). In the current study, we found that the expression rate of S100-B protein was remarkably higher in patients with PNES compared to ES patients. This finding was not in line with those observed in the study by Asadollahi et al., which may be due to the larger sample size in their study. However, it seems that further investigations are needed to confirm the true potential of S100-B for differential diagnosis of PNES and ES. 

In the present study, patients with PNES had higher levels of alpha-1-acid glycoprotein than the patients with ES patients. Alpha-1-acid glycoprotein with the molecular weight of 41-43 kD contains 45% carbohydrate in its structure (46). This glycoprotein is one of the acute-phase proteins that remarkably increases in response to various situations including infections, surgery, traumas, and myocardial infraction (47). To date, few studies have been conducted on changes in the serum level of alpha-1-acid glycoprotein in patients with epilepsy. Morita et al. showed that the serum level of alpha-1-acid glycoprotein in patients receiving enzyme-inducer anticonvulsant drugs such as carbamazepine was not significantly different from that of those receiving non-inducer drugs; but the serum level of this protein in the patients with poorly controlled seizures was significantly higher than those with well-controlled seizures (47). In another study, the serum levels of alpha-1-acid glycoprotein in patients who underwent electroconvulsive therapy were analyzed. It was reported that the serum levels of this protein were not consistently altered during electroconvulsive therapy (48). These findings show that alpha-1-acid glycoprotein can be considered as an independent biomarker for ES, which is not affected by the type of therapeutic approaches. 

## Limitations:

Low sample size and lack of easy access to patients was one of the limitations of the study.

## Conclusion:

Our results indicated that changes in serum levels of S100-β, ceruloplasmin, alpha 1-acid glycoprotein 1, and malate dehydrogenase after seizure could be introduced as potential markers to differentiate ES from PNES; however, more advanced studies are required to reach a better understanding of the underlying mechanisms. 
